# Environmental Changes Driving Shifts in the Structure and Functional Properties of the Symbiotic Microbiota of *Daphnia*

**DOI:** 10.3390/microorganisms12122492

**Published:** 2024-12-03

**Authors:** Minru You, Wenwu Yang

**Affiliations:** MOE Key Laboratory for Biodiversity Science and Ecological Engineering, School of Life Science, Fudan University, Songhu Road 2005, Shanghai 200438, China; 21210700096@m.fudan.edu.cn

**Keywords:** *Daphnia*, symbiotic microbiota, 16S rRNA, diversity, variations, transgeneration, Qinghai–Tibetan Plateau

## Abstract

Symbiotic microbiota significantly influence the development, physiology, and behavior of their hosts, and therefore, they are widely studied. However, very few studies have investigated the changes in symbiotic microbiota across generations. *Daphnia magna* originating from the Qinghai–Tibetan Plateau were cultured through seven generations in our laboratory, and the symbiotic microbiota of *D. magna* were sequenced using a 16S rRNA amplicon to analyze changes in the structure and functional properties of the symbiotic microbiota of *D. magna* from a harsh environment to an ideal environment. We detected substantial changes in the symbiotic microbiota of *D. magna* across generations. For example, the genus *Nevskia*, a member of the gamma-subclass Proteobacteria, had the highest abundance in the first generation (G1), followed by a decrease in abundance in the fourth (G4) and seventh (G7) generations. The gene functions of the microbiota in different generations of *D. magna* also changed significantly. The fourth generation was mainly rich in fatty acyl-CoA synthase, acetyl-CoA acyltransferase, phosphoglycerol phosphatase, etc. The seventh generation was mainly rich in osmotic enzyme protein and ATP-binding protein of the ABC transport system. This study confirms that the alterations in the structure and functional properties of the symbiotic microbiota of *D. magna* under changing environments are typical responses of *D. magna* to environmental changes.

## 1. Introduction

Symbiotic microbiota significantly influence development, physiology, behavior, and defense against predators in various host communities [1,2,3]. In a previous study, the symbiotic bacterium *Acetobacter pomorum* affected insulin growth factor signaling in *Drosophila*, subsequently modulating the homeostatic functions that govern the development speed, body dimensions, energy metabolism, and activity of intestinal stem cells in the host [4]. Similarly, the crustacean *Daphnia magna* requires microbiota for defense, development, and reproduction [5]. Alterations in microbial composition can occur rapidly in the face of environmental challenges, which may prompt microbes to rapidly adapt to a changing environment [6,7,8]. For instance, the composition of the symbiotic microbial community of *Nematostella vectensis* was swiftly altered by changing environmental conditions, which facilitated rapid acclimation and adaptation of the host [9]. Similarly, the microbiota of the black or Mexican molly *Poecilia sphenops* changed significantly (and in the same way) after changes in salinity levels in the environment.

*Daphnia* species (Crustacea: Cladocera) are commonly used as model organisms in ecology [10]. *D. magna* Straus, 1820 is extensively distributed in the environment. This species has been identified in North America, Europe, Asia, and Africa [11,12,13]. It has also been found within a restricted area of the Qinghai–Tibetan Plateau (QTP) [13]. It is believed that the symbiotic microbiota of *D. magna* are crucial for their extended adaptation [14,15]. The microbiota of *Daphnia* are involved in the digestion of phytoplankton [15], supplying nutrients to the host [16] and increasing their resistance to toxins [17]. A recent field survey revealed temporal variations in the gut microbiota of *D. magna*. The diversity and composition of gut microbiota varied considerably during the transfer of *Daphnia* from the lab to the lake [18]. This may be because symbiotic microbiota help *Daphnia* to cope with transient and unpredictable natural environments. However, no study has explored the transgenerational variation in the symbiotic microbiota of *Daphnia* when they are introduced from a harsh (i.e., field) environment to a laboratory with an ideal environment.

The Qinghai–Tibetan Plateau, located in China, is considered the world’s highest elevated plateau [19]. It has an extremely harsh environment, with a cold and dry climate, thin oxygen, and a high intensity of UV radiation [20,21]. For the majority of *Daphnia* species, a water temperature ranging from 15 to 22 degrees is optimal for asexual reproduction. The laboratory creates favorable conditions for the growth and reproduction of *Daphnia* by providing suitable water quality, temperature, light, nutrition, and culture management. We assumed that *Daphnia* living in this special environment of the Qinghai–Tibetan Plateau acquired special symbiotic microbes. Thus, a drastic reduction in both microbial diversity and abundance was expected when *Daphnia* were translocated from the ponds/lakes of the Qinghai–Tibetan Plateau to a laboratory culture with an ideal environment.

## 2. Materials and Methods

### 2.1. Research Organism

**Figure 1:**
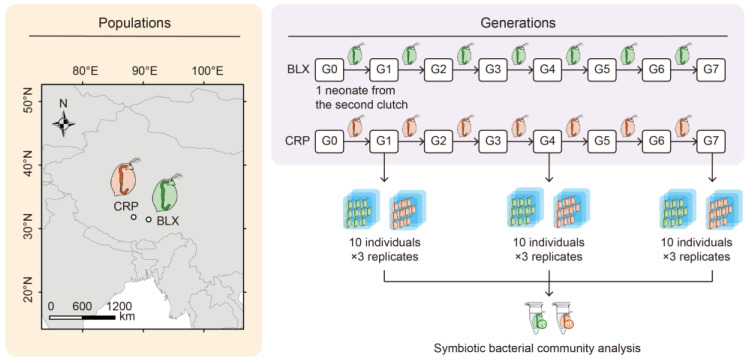
Experimental design for cultivation of *D. magna*, isolated from BLX and CRP ponds on Qinghai–Tibetan Plateau. Here, G0 represents original individuals isolated from each pond; G1 represents first generation; G4 represents fourth generation; and G7 represents seventh generation.

*Daphnia magna* used in this study was originally isolated from two ponds of the Qinghai–Tibetan Plateau: the BLX (31°23′ N, 90°53′ E) and CRP (31°47′ N, 88°26′ E) ponds (Figure 1). It was identified through a phylogenetic analysis based on the sequencing of the CO1 gene [22]. The stock cultures of *D. magna* were maintained in the lab in COMBO medium [23], stored at 20 °C with a 16:8 h light/dark cycle, and fed with the unicellular algae *Ankistrodesmus falcatus* three times a week.

### 2.2. Cultivation of Axenic Ankistrodesmus Falcatus

Axenic *Ankistrodesmus falcatus* was cultured by adding 20 μL of microalgae inoculum into a sterile Erlenmeyer flask filled with 200 mL of autoclaved COMBO medium. Cultures were incubated for 5 days at 20 °C under a 16:8 h light/dark cycle on a shaking plate. The cells were then collected, thoroughly cleaned twice with diethyl pyrocarbonate (DEPC) water, and resuspended into filtered COMBO medium. Before use, the harvested cells were kept at 4 °C. The axenic characteristic of *A. falcatus* was verified by using LB-medium agar plates.

### 2.3. Experimental Setup

A single adult *D. magna* female was isolated from each pond as Generation 0 (G0; Figure 1) and transferred to the laboratory. A single neonate from the second clutch produced by each mother (G0) was randomly selected and cultured in a single beaker to obtain the next parthenogenetic generation. The process was continued until Generation 7 (G7). Ten adult *D. magna* females from the first (G1), fourth (G4), and seventh (G7) generations were collected and pooled for an analysis of their associated microbiota. For each pond, this entire process was replicated three times (i.e., three adult *D. magna* females were randomly selected as three biological replicates representing G0), resulting in 18 samples in this study. All these experiments were conducted in separate culturing tanks and under sterile conditions (except G0 to G1). Pond water was used as a medium to culture *D. magna* from G0 to G1. From G1 to G7, *D. magna* were cultured in the sterile COMBO medium. The medium was changed every other day, and *D. magna* were fed with sterile *A. falcatus* daily.

### 2.4. Microbiota Analysis

*D. magna* were starved in beakers for a day and then rinsed three times with DEPC-treated water in Eppendorf tubes. Total DNA was extracted utilizing the PowerSoil DNA Separation Kit (MO BIO, Carlsbad, CA, USA) according to the manufacturer’s protocol. The purity and concentration of the DNA were measured using 1% agarose gel electrophoresis. The pure DNA was diluted to 1 ng/μL using DEPC water. Subsequently, the highly variable V4 region of the 16S rRNA gene was amplified through polymerase chain reaction (PCR) using the primers 515F and 806R [24]. The 5′ end of each primer had a barcode. The PCR mix contained 0.2 mM of each primer, 10 ng of template DNA, and 30 μL of PCR Master Mix (New England Biolabs’ Phusion Hi-Fi). The PCR procedure included several steps. It began with preliminary denaturation at 98 °C for 1 min, followed by 30 thermal cycles. Each cycle consisted of a denaturation phase at 98 °C for 10 s, an annealing phase at 50 °C for 30 s, and an extension phase at 72 °C for another 30 s. Finally, there was a concluding extension step at 72 °C that lasted for 5 min. The PCR products obtained from each sample were purified using a Qiagen Gel Extraction Kit (Qiagen, Hilden, Germany). Subsequently, an equimolar library was prepared by pooling the normalized amplicon concentrations using the Applied Biosystems SequalPrep Normalization Plate. The amplicons were sequenced on an Illumina Miseq platform using a v2 PE500 kit and custom primers (San Diego, CA, USA). The sequencing process provided 2 × 250 bp paired-end reads.

Subsequently, raw reads were filtered and trimmed using DADA2 (Version 1.34.0) [25]. The fastqPairedFilter (for paired reads) and fastqFilter (for forward reads only) functions were employed to remove the sequences featuring Ns or more than two predicted errors, as well as to prune the first 20 nucleotides and the last 10 (for forward reads) or 10–50 (for reverse reads) nucleotides based on the quality. DADA2 was then employed with the default parameters for dereplication and with the merging of paired-end reads to eliminate the chimeras. Finally, amplicon sequence variants (ASVs) were generated using DADA2. Amplicon sequence variants with an occurrence of <1% were pooled in the “others” category. Taxonomic classification of these ASVs was conducted utilizing the Silva138.1 database within QIIME2 (Version QIIM2-202006) [26].

The α-diversity indices (Shannon index and abundance-based coverage estimator (ACE) index) were calculated for each sample utilizing the “vegan” package (Version 2.6-8) [27] in R (Version 4.1.0) [28]. The α-diversity indices of the symbiotic microbiota of *D. magna* were compared across three generations (i.e., G1, G4, and G7), and the generational effects on α-diversity were assessed using an analysis of variance (ANOVA) in GraphPad (Version 10.4.0). To investigate the differences between the compositions of symbiotic microbiota of *D. magna* (β-diversity) across different generations, the weighted UniFrac distances were calculated and visualized through principal coordinate analysis (PCoA), employing the phyloseq package (Version 1.50.0) in R (Version 4.1.0) [29]. This approach facilitated the plotting of the weighted distances. A permutational multivariate analysis of variance (MANOVA) was conducted to assess the generational effects on β-diversity in GraphPad. Finally, a Linear discriminant analysis Effect Size (LEfSe) analysis was conducted using LEfSe V 1.0 software to identify and differentiate the taxa showing significant changes in abundance across the three generations [30]. Furthermore, Kruskal–Wallis and Wilcoxon rank-sum tests were conducted in the Linear discriminant analysis Effect Size (LEFSe) analysis to analyze the sample data, facilitating the classification of taxa. Any taxon with a linear discriminant analysis (LDA) effect size > 4 and a *p*-value < 0.05 was considered to differ significantly in abundance across generations. Further, PICRUSt2 software (Version 2.1.2-b) was used to predict the functions of the microbial communities.

## 3. Results

### 3.1. Sequencing Results

A total of 18 symbiotic microbiota samples from three generations of *D. magna* (two ponds: BLX and CRP) were collected. The total DNA of symbiotic microbiota was extracted and sequenced, targeting the V4 segment of the 16S rRNA gene, which resulted in the generation of a total of 1,480,359 raw sequences (Table 1). After quality filtering and chimera removal, a total of 1,259,280 high-quality sequences with an average 253 bp length were derived, representing 85% of the eligible sequences. The dataset encompassed an average of 69,960 sequences per sample, ranging from 61,169 to 77,536 (Table 1). To mitigate the bias stemming from sequencing depth, the sequence count was equalized by random subsampling to match the sample with the minimal sequence count. The high coverage values (100%) across all samples indicated adequate sequencing depth. Additionally, the values of Q20 (>99%) and Q30 (>97%; Table 1) in each sample suggested that the sequencing data were of high quality.

### 3.2. Changes in Symbiotic Microbiota Across Generations

**Figure 2:**
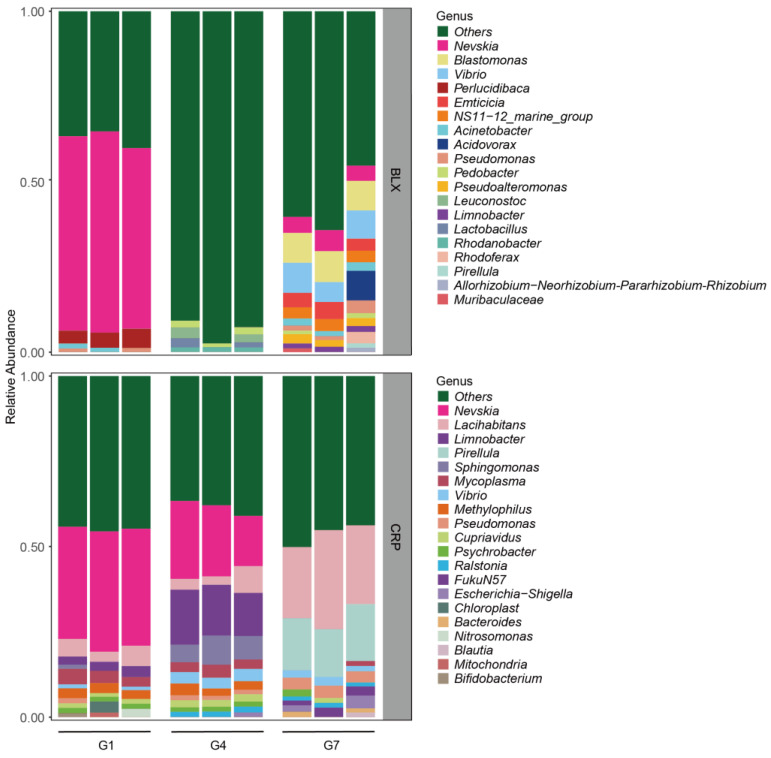
Temporal shifts in the symbiotic microbial communities across three generations (G1, G4, and G7) of *D. magna* from two ponds on the Qinghai–Tibetan Plateau. The distinct microbial genera are indicated by different colors. Amplicon sequence variants (ASVs) with an occurrence of <1% were pooled in the “others” category. The gray bar on the right side shows the name of the source pond.

The results revealed substantial changes in the symbiotic microbial communities across three generations of *D. magna* from both ponds (Figure 2). Specifically, the symbiotic microbial communities of *D. magna* from the BLX pond were mainly dominated by three genera: *Nevskia* (56.4% ± 3.1%SD), *Perlucidibaca* (4.6% ± 1.0%SD), and *Acinetobacter* (0.9% ± 0.8%SD) in G1. The dominant genera in G7 were *Blastomonas* (8.8% ± 0.2%SD), *Vibrio* (7.6% ± 1.6%SD), and *Nevskia* (5.1% ± 0.9%SD). The top genera in G4 were *Leuconostoc* (1.9% ± 1.7%SD), *Pedobacter* (1.7% ± 0.5%SD), *Lactobacillus* (1.4% ± 1.3%SD), and *Rhodanobacter* (1.4% ± 0.1%SD); however, none of these genera showed a very high abundance in G4 (Figure 2 and Table 2). Interestingly, *Nevskia* dominated in G1, but disappeared in G4, and re-appeared in G7 (Figure 2 and Table 2). *Perlucidibaca* was the second most abundant genus in G1, but it was not present in G4 and the G7 (Figure 2 and Table 2). Furthermore, 16 genera, including *Blastomonas*, *Vibrio*, *Acidovorax*, *Emticicia*, *NS11-12_marine_group*, *Rhodoferax*, *Pseudoalteromonas*, *Acinetobacter*, *Limnobacter*, *Pirellula*, *Allorhizobium*-Neo, *Muribaculaceae*, *Leuconostoc*, *Lactobacillus*, *Pedobacter*, and *Rhodanobacter* were detected in G7. Four of these genera, i.e., *Leuconostoc*, *Lactobacillus*, *Pedobacter*, *Rhodanobacter*, were not present in G1 (Figure 2 and Table 2). The symbiotic microbial communities of *D. magna* from the CRP pond were mainly dominated by three genera, *Nevskia* (34.2% ± 1.2%SD), *Lacihabitans* (4.7% ± 1.6%SD), and *Mycoplasma* (3.6% ± 0.9%SD), in G1. The three dominant genera in G4 were *Nevskia* (19.5% ± 4.2%SD), *Limnobacter* (14.6% ± 1.8%SD), and *Sphingomonas* (6.8% ± 1.7%SD), while the dominant genera in G7 were *Lacihabitans* 24.3% ± 4.2%SD), *Pirellula* (15.3% ± 1.4%SD), and *Pseudomonas* (3.4% ± 0.2%SD) (Figure 2 and Table 3). Interestingly, *Nevskia* dominated in G1 and G4, but was not present in G7 (Figure 2 and Table 3). *Perlucidibaca* was the second most abundant genus in G1, but disappeared in G4 and G7 (Figure 2 and Table 3). Five genera (*Pirellula*, *Escherichia-Shigella*, *FukuN57*, *Bacteroides*, and *Blautia*), which were not detected in G1 or G4, emerged in G7 (Figure 2 and Table 3).

**Figure 3:**
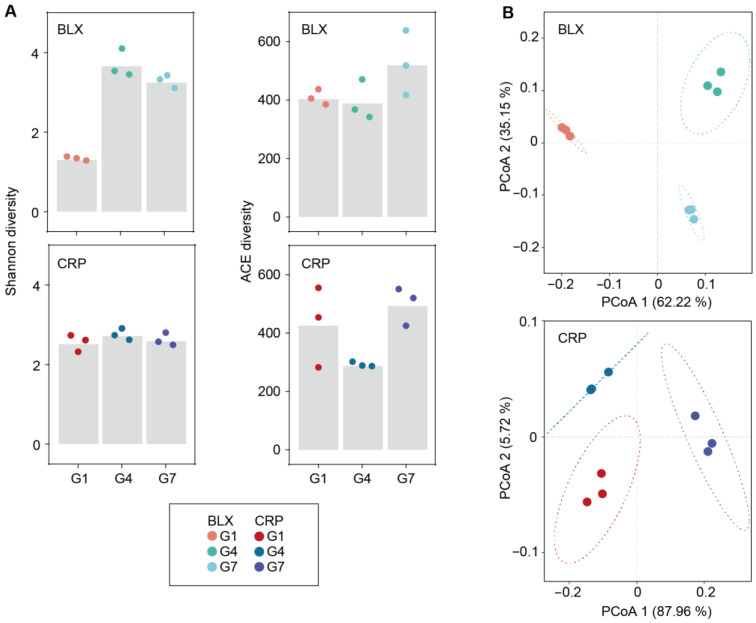
Temporal changes in symbiotic microbial richness and diversities across three generations (G1, G4, and G7) of *D. magna* from two different ponds: (**A**) α−diversity (Shannon and ACE indexes) of symbiotic microbiota of *D. magna* across three generations. Bars represent mean values, while points represent real values. (**B**) Principal coordinate analysis (PCoA) of symbiotic microbiota across three generations of *D. magna*, based on weighted UniFrac distance.

A significant trend in α-diversity (Shannon index) was detected in the clone from BLX, with the Shannon index of G4 or G7 being higher than that of G1. However, this trend was not observed for the CRP clone (Figure 3A and Table 4 and Table S1). The principal coordinate analysis (PCoA) showed clear separation of samples along the first two axes, with Principal Component 1 accounting for 65.22% and 87.96% of the total variations in BLX and CRP, respectively. Principal Component 2 accounted for 35.15% and 5.72% of the total variations in BLX and CRP, respectively (Figure 3B). The distinct clustering of samples indicated significant differences between the symbiotic microbial communities of three generations of *D. magna* (Figure 3B). Furthermore, β-diversity analysis based on the weighted UniFrac metric revealed that the majority of the differences between the symbiotic microbial community compositions could be attributed to the generational effects in both populations (permutation MANOVA: *p* = 0.01 for BLX and *p* < 0.01 for CRP; Figure 3B and Table S2).

**Figure 4:**
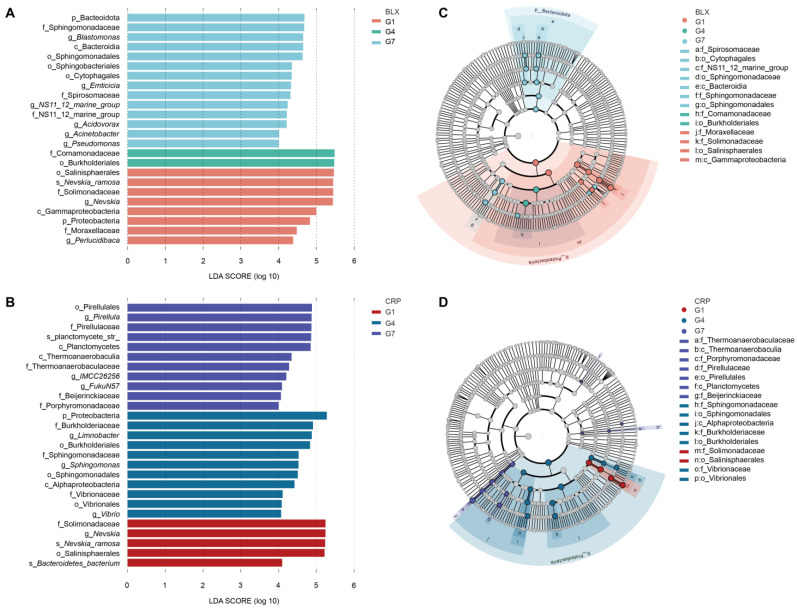
Linear discriminant analysis Effect Size (LEFSe) analysis showing statistically significant microbial biomarkers across three generations of *D. magna*. The histograms show the species that exceeded the linear discriminant analysis (LDA) score threshold of 4 in (**A**) BLX and (**C**) CRP, indicating variations in their abundance across different generations of *D. magna*. The bar lengths correspond to the impact magnitude of each taxon (LDA score). The cladograms were derived from the LEfSe analysis of taxa, with different abundances across the three generations of *D. magna*, in (**B**) BLX and (**D**) CRP. The concentric circles starting from the center depict taxonomic ranks from phylum to genus or species. The size of each circle at varying taxonomic levels signifies the relative abundance of corresponding taxa, with uniform gray shading showing no significant variation. Notable taxonomic differences between generations are indicated by specific colors. Nodes denote key microbial species, while circle diameters reflect the relative abundance of species. Prefixes denote classification levels: ‘p’ for phylum, ‘c’ for class, ‘o’ for order, ‘f’ for family, ‘g’ for genus, and ‘s’ for species.

The Linear discriminant analysis Effect Size (LEfSe) analysis revealed shifts in the symbiotic microbial communities across the three generations of *D. magna* in both BLX and CRP (Figure 4). A total of 14 (or 11), 2 (11), and 8 (5) biomarkers specific to G1, G4, and G7, respectively, were detected in BLX (Figure 4A,C; Table S3) to distinguish the taxonomic differences across the generations. In CRP, 11, 11, and 5 biomarkers were detected for G1, G4, and G7, respectively. As shown in the cladograms (Figure 4B,D), the taxonomic distributions further confirmed that the high abundance of specific microbial taxa was associated with specific generations. At the genus level in BLX, there was a greater abundance of *Nevskia* and *Perlucidibaca* in G1, while *Blastomonas*, *Emticicia*, *NS11_12_marine_group*, *Acidovorax*, *Acinetobacter*, and *Pseudomonas* were more abundant in G7. However, no genus had greater abundance in G4 (Figure 4A and Table S3). In CRP, *Nevskia* has the highest abundance in G1, while *Limnobacter*, *Sphingomonas*, and *Vibrio* were more abundant in G4. *Pirellula*, *IMCC26256*, and *FukuN57* showed high abundance in G7 (Figure 4C and Table S4). At the species level in BLX, *Nevskia_ramosa* was enriched in G1, while its abundance decreased in G4 and G7 (Figure 4A and Table S3). In CRP, *Nevskia_ramosa* and *Bacteroidetes*_bacterium were enriched in G1, while *planctomycete_str* was enriched in G7. However, no species showed a very high abundance in G4 (Figure 4C and Table S4). Proteobacteria contributed significantly to the differentiation of microbiota across the three generations in both BLX and CRP (Figure 4B,D). Specifically, Salinisphaerales, an order of Proteobacteria, was abundant in G1. However, the abundance of this order decreased substantially in G4 and G7 (Figure 4B,D).

**Figure 5:**
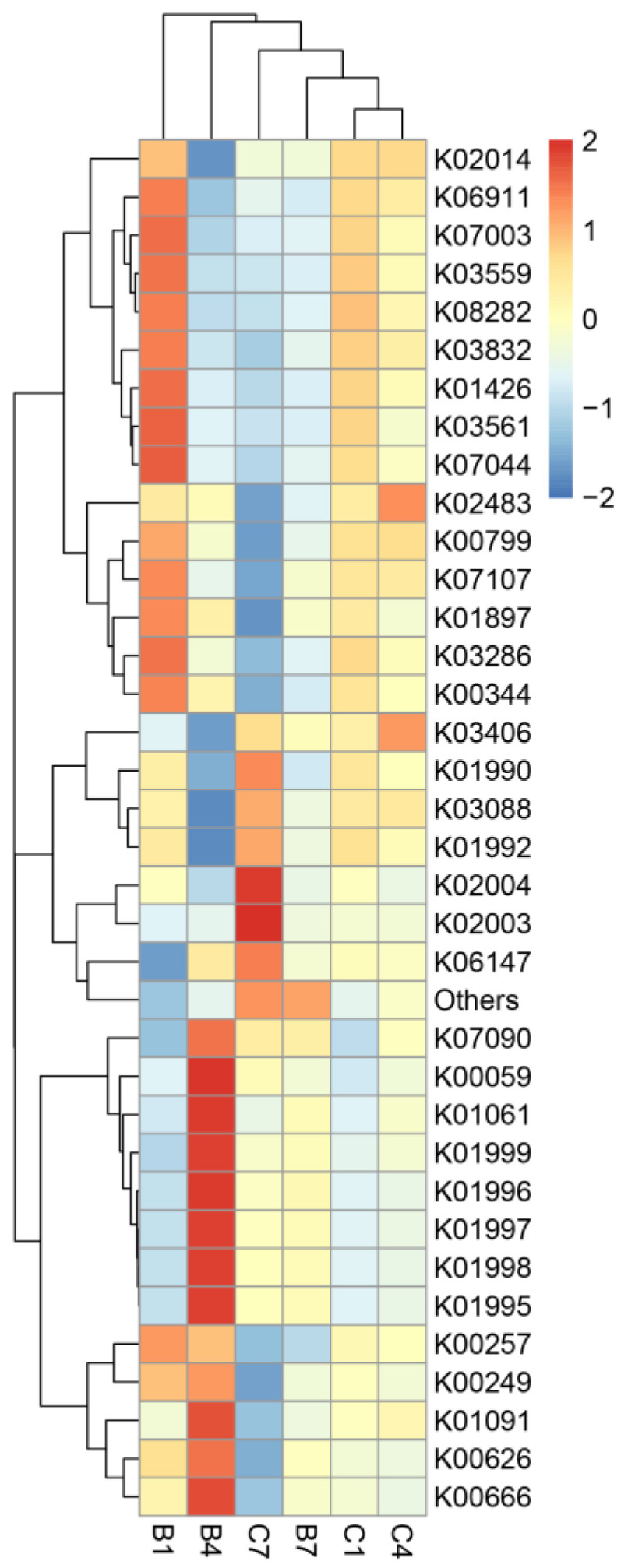
A PICRUSt2 function annotation clustering heatmap showing the function annotation clustering of symbiotic microbiota across three generations of *D. magna*. Different generations are shown on horizontal coordinates, while functional annotation information is shown on vertical coordinates. The functional dendrogram situated on the left side represents a cluster tree. The heatmap shows the Z-scores derived from the normalized functional abundance of each entry. Specifically, the Z score of a sample within a particular category is calculated by dividing the deviation of its relative abundance from the mean relative abundance within the category by the standard deviation across all samples within that category.

Based on the PICRUSt2 functional annotations and prevalence data in the database, the 35 most abundant functions based on their prevalence across samples were chosen to create a heatmap. Clustering was performed at various functional levels to visualize the data. The KO database was used to predict the functions of genes in the symbiotic microbiota of different generations of *D. magna*. The functional annotation revealed that G4 was mainly enriched with fatty acyl-CoA synthase, acetyl-CoA acyltransferase, phosphoglycerol phosphatase, branched-chain amino acid transporter ATPase subunit, branched-chain amino acid permease protein, branched-chain amino acid transporter substrate-binding subunit, 3-oxyacyl carrier protein reductase, etc. On the other hand, ABC transporter osmotic enzyme subunit and ABC transporter ATPase subunit were more prevalent functions in G7 (Figure 5).

## 4. Discussion

It is well recognized that symbiotic microbiota can mediate the acclimatization and adaptation of their host to changing environments [31,32,33]. In the present study, the relative abundance of the dominant microbial taxa changed dynamically across generations after the transfer of their host *D. magna* from ponds on the Qinghai–Tibetan Plateau (harsh environment) to laboratory conditions (ideal environment). This suggests that the reconfiguration of symbiotic microbial communities may help *Daphnia* to adapt to shifts in the environment. Nevertheless, these results should be interpreted carefully. Although the “earliest” microbiota composition (G1) was established by cultivating *D. magna* in pond water collected from the Qinghai–Tibetan Plateau, the composition of microbiota might have already changed during the rapid transfer of *D. magna* to the laboratory due to transient changes in environmental factors. Therefore, further research is needed to investigate the composition of the natural symbiotic microbiota of *Daphnia* on the Qinghai–Tibetan Plateau.

In this study, most microbial ASVs across the three different generations of *D. magna* from both ponds belonged to Proteobacteria, including the *Nevskia*, *Perlucidibaca*, *Blastomonas*, *Acidovorax*, *Acinetobacter*, *Pseudomonas*, *Limnobacter*, *Sphingomonas*, *Vibrio*, and *FukuN57* genera. This was in line with previous studies, which reported Proteobacteria as the most abundant group of microbial symbionts of *Daphnia* in both laboratory cultures and field samples [34,35]. A previous study has already shown the beneficial effects of the members of Proteobacteria on *Daphnia*, especially in terms of helping *Daphnia* to cope with various environmental stresses [36]. *Sphingomonas*, *Pseudomonas*, and *Phenylbacterium* can degrade microcystin, the main toxin generated by cyanobacteria, thus improving the tolerance of *Daphnia* to cyanobacteria [17]. Also, *Limnohabitans* (belonging to Proteobacteria) is believed to contribute to the fertility and population growth of *Daphnia* [37] by improving the ingestion and assimilation of food [38]. Considering their positive effects on the fitness of host, we assumed that the members of Proteobacteria are indispensable in the adaptation of *D. magna* to environmental shifts.

Interestingly, the genus *Nevskia*, a member of the gamma-subclass of Proteobacteria, showed the highest abundance in G1, which subsequently decreased in G4 and G7 of *D. magna* from both ponds. *Nevskia* bacterium typically lives at the air–water interface of freshwater ecosystems [39]. In this habitat, it must have high tolerance to ultraviolet (UV) radiation from the sun. *Nevskia* has an effective DNA repair mechanism against the damage caused by UV radiation [40]. Interestingly, *Nevskia* possesses a notably higher proportion of guanosine and cytosine (typically exceeding 67%), which could be potentially helpful in mitigating UV-induced DNA damage through the prevention of thymidine dimerization [41]. Here, the microbiota of *Daphnia* from both ponds were dominated by *Nevskia* in G1, suggesting that this genus might have helped *Daphnia* (presenting in the carapace) to cope with the harsh environment (UV radiation) on the Qinghai–Tibetan Plateau. Once the host *Daphnia* was transferred to the ideal environment of the laboratory culture, the abundance of *Nevskia* significantly declined across generations in the absence of UV exposure.

Additionally, *Leuconostoc* and *Lactobacillus* appear in G4 and then disappear. *Leuconostoc* and *lactobacillus* may be probiotic and may have anti-pathogenic activity that may have helped in their adaptation to new environments [42,43,44,45]

The abundance of the members of the order Burkholderiales increased significantly in G4 of *D. magna* from both ponds, suggesting that these microbes might have helped *D. magna* to adapt to the laboratory environment. Burkholderiales is one of the most abundant microbial taxa within a natural environment, and their ability to adapt to fluctuating conditions has enabled them to inhabit a variety of ecological niches [46,47]. Burkholderia spp. has been reported to trigger systemic resistance, thus enhancing the resistance of their hosts against abiotic stress [47]. For example, Burkholderia phytofirmans strain PsJN improved the heat tolerance of potatoes [48]. Additionally, Burkholderia cepacia showed strong phenotypic plasticity, which enabled it to thrive under diverse environmental conditions [49]. These findings suggest that the members of Burkholderiales can serve as effective microbial shields for *Daphnia* within particular environmental niches, inducing the metabolism of the host in response to the changing environment [50].

The local environment typically emerges as a crucial factor shaping the symbiotic microbial community of host [51]. Indeed, the symbiotic microbial communities of freshwater zooplankton exhibit good adaptability and are significantly altered by the environment [52]. In previous research, we observed that the composition of the symbiotic microbiota of *Daphnia* in laboratory conditions was not consistently stable. Perhaps the changes in the composition of the microbiota are due to the stable environment in the laboratory, unlike the harsh environment where the *Daphnia* were initially sourced from. The laboratory setting promoted an increase in the prevalence of ASVs that were more suited to the new conditions. This finding demonstrates the considerable variability in the microbial community associated with *Daphnia*, particularly at finer taxonomic levels. Previous studies have also documented the high flexibility of microbial communities in *Daphnia* [53,54]. For example, previous researchers cultivated *D. magna* clones under varying temperatures for three successive generations and observed substantial differences in their microbial communities [55]. Other ecological factors, such as diet [56], environment [53] and antibiotic exposure [35], also exerted a significant influence on the composition of symbiotic microbiota in *Daphnia*. This flexibility might be advantageous to *Daphnia*, enabling the expansion of their ecological niches and helping them to adapt to new environmental conditions.

In addition, microbial gene functions varied significantly across different generations of *Daphnia*. The functional annotation revealed that G4 was mainly enriched with fatty acyl-CoA synthase, acetyl-CoA acyltransferase, phosphoglycerol phosphatase, branched-chain amino acid transporter ATPase subunit, branched-chain amino acid permease protein, branched-chain amino acid transporter substrate-binding subunit, 3-oxyacyl carrier protein reductase, etc. On the other hand, ABC transporter osmotic enzyme subunit and ABC transporter ATPase subunit were more prevalent functions in G7.

## 5. Conclusions

In conclusion, this study provided further evidence for the claim that the structure and functional properties of the symbiotic microbial communities of *D. magna*, which survive under a harsh field environment, change significantly after their transfer to an ideal environment in the laboratory. This suggests that structural alterations and functional properties of symbiotic microbial communities in changing environments are common features of *D. magna* in response to environmental changes. This finding not only provides new ideas to explore the resistance of host and symbiotic microbiota to environmental stress, but also lays a foundation to investigate the tolerance mechanism of organisms in adverse environments, as well as the homeostatic regulation mechanism of symbiotic microbial communities associated with them.

## Figures and Tables

**Table 1:** The metadata for the symbiotic microbiota of *D. magna* across generations (G1, 4, and 7) from the “BLX” and “CRP” ponds with the base length, quality scores, and content of GC and Effective Tags. Raw PE: the original PE reads; Combined: the spliced Tags sequence; Qualified: the sequence of Raw Tags after filtering low-quality and short-length sequences; Nochime: the final Tags sequence after filtering chimeras, i.e., Effective Tags; Base: the number of bases of the final Effective Tags; Base: the number of bases in the final Effective Tags; AvgLen: the average length of the Effective Tags; Q20 and Q30: the percentage of bases in the Effective Tags with base quality values greater than 20 (sequencing error rate less than 1%) and 30 (sequencing error rate less than 0.1%); GC (%): the number of GC bases in the Effective Tags; Effective(%): the percentage of the number of Effective Tags among the number of Raw PE reads.

Sample Name	Raw PE	Combined	Qualified	Nochime	Base (nt)	AvgLen (nt)	Q20	Q30	GC%	Effective%
BLX-G1.1	83,200	82,788	82,566	74,012	18,725,658	253	99.34	97.49	54.37	88.96
BLX-G1.2	89,087	87,990	87,424	77,536	19,616,285	253	99.29	97.37	54.35	87.03
BLX-G1.3	79,898	79,652	79,472	69,472	17,576,179	253	99.36	97.55	54.36	86.95
BLX-G4.1	70,345	68,448	68,224	63,773	16,133,089	253	99.49	98.08	54.23	90.66
BLX-G4.2	80,580	79,809	79,604	74,448	18,832,339	253	99.52	98.14	54.43	92.39
BLXG4.3	80,695	79,482	79,208	74,430	18,824,748	253	99.5	98.08	54.29	92.24
BLXG7.1	87,116	85,198	84,891	61,169	15,481,354	253	99.18	97.04	53.49	70.22
BLXG7.2	88,874	88,368	88,212	66,341	16,786,801	253	99.31	97.42	53.61	74.65
BLXG7.3	84,465	82,614	82,317	67,282	17,026,164	253	99.29	97.38	53.48	79.66
CRPG-1.1	79,628	78,796	78,717	67,911	17,189,725	253	99.61	98.43	53.44	85.29
CRPG1.2	87,199	86,575	86,511	75,022	18,981,147	253	99.63	98.48	53.87	86.04
CRPG 1.3	79,676	79,114	79,058	67,761	17,150,369	253	99.62	98.44	53.44	85.05
CRP-G4.1	82,658	81,468	81,393	71,177	18,024,238	253	99.64	98.52	53.39	86.11
CRP-G4.2	79,841	78,507	78,456	65,134	16,484,792	253	99.62	98.46	53.3	81.58
CRP-G4.3	84,457	82,625	82,552	72,148	18,293,095	254	99.62	98.43	53.05	85.43
CRP-G7.1	73,089	71,654	71,586	65,504	16,608,432	254	99.61	98.43	52.61	89.62
CRP-G7.2	85,040	83,639	83,560	74,237	18,830,649	254	99.59	98.34	52.18	87.3
CRP-G7.3	84,511	83,075	83,002	71,923	18,242,441	254	99.62	98.44	52.48	85.1

**Table 2:** Relative abundance and taxonomy details of bacterial taxa of symbiotic microbiota of *D. magna* from “BLX” pond. Relative abundances for experimental samples grouped at genus rank at 1% threshold.

Taxonomy	BLX -G1.1	BLX -G1.2	BLX -G1.3	BLX -G4.1	BLX -G4.2	BLX -G4.3	BLX -G7.1	BLX -G7.2	BLX -G7.3	Tax_Detail
*Nevskia*	0.57	0.59	0.53	0	0	0	0.05	0.06	0.04	k_Bacteria;p_Proteobacteria;c_Gammaproteobacteria; o_Salinisphaerales;f_Solimonadaceae;g_*Nevskia*;
*Blastomonas*	0	0	0	0	0	0	0.09	0.09	0.09	k_Bacteria;p_Proteobacteria;c_Alphaproteobacteria; o_Sphingomonadales;f_Sphingomonadaceae; g_*Blastomonas*;
*Vibrio*	0	0	0	0	0	0	0.09	0.06	0.08	k_Bacteria;p_Proteobacteria;c_Gammaproteobacteria; o_Vibrionales;f_Vibrionaceae;g_*Vibrio*;
*Acidovorax*	0	0	0	0	0	0	0	0	0.09	k_Bacteria;p_Proteobacteria;c_Gammaproteobacteria; o_Burkholderiales;f_Comamonadaceae;g_*Acidovorax*;
*Perlucidibaca*	0.04	0.04	0.06	0	0	0	0	0	0	k_Bacteria;p_Proteobacteria;c_Gammaproteobacteria; o_Pseudomonadales;f_Moraxellaceae;g_*Perlucidibaca*;
*Emticicia*	0	0	0	0	0	0	0.04	0.05	0.04	k_Bacteria;p_Bacteroidota;c_Bacteroidia;o_Cytophagales;f_Spirosomaceae;g_*Emticicia*;
*Pseudomonas*	0.01	0	0.01	0	0	0	0.01	0.01	0.04	k_Bacteria;p_Proteobacteria;c_Gammaproteobacteria; o_Pseudomonadales;f_Pseudomonadaceae; g_*Pseudomonas*;
*NS11-12_marine_* *group*	0	0	0	0	0	0	0.03	0.04	0.03	k_Bacteria;p_Bacteroidota;c_Bacteroidia; o_Sphingobacteriales;f_NS11-12_marine_group;g_*NS11-12_marine_group*;
*Rhodoferax*	0	0	0	0	0	0	0	0	0.03	k_Bacteria;p_Proteobacteria;c_Gammaproteobacteria; o_Burkholderiales;f_Comamonadaceae;g_*Rhodoferax*;
*Leuconostoc*	0	0	0	0.03	0	0.02	0	0	0	k_Bacteria;p_Firmicutes;c_Bacilli;o_Lactobacillales; f_Leuconostocaceae;g_*Leuconostoc*;
*Pseudoalteromonas*	0	0	0	0	0	0	0.03	0.02	0.02	k_Bacteria;p_Proteobacteria;c_Gammaproteobacteria; o_Alteromonadales;f_Pseudoalteromonadaceae; g_*Pseudoalteromonas*;
*Lactobacillus*	0	0	0	0.03	0	0.02	0	0	0	k_Bacteria;p_Firmicutes;c_Bacilli;o_Lactobacillales; f_Lactobacillaceae;g_*Lactobacillus*;
*Acinetobacter*	0.01	0.01	0	0	0	0	0.02	0.01	0.06	k_Bacteria;p_Proteobacteria;c_Gammaproteobacteria; o_Pseudomonadales;f_Moraxellaceae;g_*Acinetobacter*;
*Pedobacter*	0	0	0	0.02	0.01	0.02	0.01	0	0.01	k_Bacteria;p_Bacteroidota;c_Bacteroidia; o_Sphingobacteriales;f_Sphingobacteriaceae;g_*Pedobacter*;
*Limnobacter*	0	0	0	0	0	0	0.01	0.02	0.02	k_Bacteria;p_Proteobacteria;c_Gammaproteobacteria; o_Burkholderiales;f_Burkholderiaceae;g_*Limnobacter*;
*Rhodanobacter*	0	0	0	0.01	0.01	0.01	0	0	0	k_Bacteria;p_Proteobacteria;c_Gammaproteobacteria; o_Xanthomonadales;f_Rhodanobacteraceae; g_*Rhodanobacter*;
*Pirellula*	0	0	0	0	0	0	0	0	0.01	k_Bacteria;p_Planctomycetota;c_Planctomycetes; o_Pirellulales;f_Pirellulaceae;g_*Pirellula*;
*Allorhizobium-Neo*	0	0	0	0	0	0	0	0	0.01	k_Bacteria;p_Proteobacteria;c_Alphaproteobacteria; o_Rhizobiales;f_Rhizobiaceae; g_*Allorhizobium-Neorhizobium-Pararhizobium-Rhizobium*;
*Muribaculaceae*	0	0	0	0	0	0	0.01	0	0	k_Bacteria;p_Bacteroidota;c_Bacteroidia;o_Bacteroidales; f_Muribaculaceae;g_*Muribaculaceae*;

**Table 3:** Relative abundance and taxonomy details of bacterial taxa of symbiotic microbiota of *D. magna* from “CRP” pond. Relative abundances for experimental samples grouped at genus rank at 1% threshold.

Taxonomy	CRP -G1.1	CRP -G1.2	CRP -G1.3	CRP -G4.1	CRP -G4.2	CRP -G4.3	CRP -G7.1	CRP -G7.2	CRP -G7.3	Tax_Detail
*Nevskia*	0.33	0.35	0.34	0.23	0.21	0.15	0.00	0.00	0.00	k_Bacteria;p_Proteobacteria;c_Gammaproteobacteria; o_Salinisphaerales;f_Solimonadaceae;g_*Nevskia*;
*Lacihabitans*	0.05	0.03	0.06	0.03	0.02	0.08	0.21	0.29	0.23	k_Bacteria;p_Bacteroidota;c_Bacteroidia; o_Cytophagales;f_Spirosomaceae;g_*Lacihabitans*;
*Pirellula*	0.00	0.00	0.00	0.00	0.00	0.00	0.15	0.14	0.17	k_Bacteria;p_Planctomycetota;c_Planctomycetes; o_Pirellulales;f_Pirellulaceae;g_*Pirellula*;
*Limnobacter*	0.02	0.03	0.03	0.16	0.15	0.13	0.00	0.00	0.00	k_Bacteria;p_Proteobacteria;c_Gammaproteobacteria; o_Burkholderiales;f_Burkholderiaceae;g_*Limnobacter*;
*Sphingomonas*	0.01	0.00	0.00	0.05	0.09	0.07	0.00	0.00	0.00	k_Bacteria;p_Proteobacteria;c_Alphaproteobacteria; o_Sphingomonadales;f_Sphingomonadaceae; g_*Sphingomonas*;
*Mycoplasma*	0.04	0.04	0.03	0.03	0.04	0.03	0.00	0.00	0.01	k_Bacteria;p_Firmicutes;c_Bacilli;o_Mycoplasmatales; f_Mycoplasmataceae;g_*Mycoplasma*;
*Escherichia-Shigella*	0.00	0.00	0.00	0.00	0.00	0.01	0.02	0.00	0.04	k_Bacteria;p_Proteobacteria;c_Gammaproteobacteria;o_Enterobacterales;f_Enterobacteriaceae; g_*Escherichia-Shigella*;
*Pseudomonas*	0.02	0.00	0.00	0.01	0.01	0.01	0.03	0.04	0.03	k_Bacteria;p_Proteobacteria;c_Gammaproteobacteria;o_Pseudomonadales;f_Pseudomonadaceae; g_*Pseudomonas*;
*Vibrio*	0.01	0.00	0.01	0.03	0.03	0.04	0.02	0.03	0.02	k_Bacteria;p_Proteobacteria;c_Gammaproteobacteria;o_Vibrionales;f_Vibrionaceae;g_*Vibrio*;
*Methylophilus*	0.03	0.03	0.03	0.03	0.02	0.02	0.00	0.00	0.00	k_Bacteria;p_Proteobacteria;c_Gammaproteobacteria;o_Burkholderiales;f_Methylophilaceae; g_*Methylophilus*;
*Chloroplast*	0.00	0.03	0.00	0.00	0.00	0.00	0.00	0.00	0.00	k_Bacteria;p_Cyanobacteria;c_Cyanobacteriia; o_Chloroplast;f_Chloroplast;g_*Chloroplast*;
*FukuN57*	0.00	0.00	0.00	0.00	0.00	0.00	0.01	0.03	0.03	k_Bacteria;p_Proteobacteria;c_Alphaproteobacteria; o_Rhizobiales;f_Beijerinckiaceae;g_*FukuN57*;
*Nitrosomonas*	0.00	0.00	0.02	0.00	0.00	0.00	0.00	0.00	0.00	k_Bacteria;p_Proteobacteria;c_Gammaproteobacteria;o_Burkholderiales;f_Nitrosomonadaceae; g_*Nitrosomonas*;
*Cupriavidus*	0.01	0.01	0.01	0.02	0.02	0.02	0.00	0.01	0.00	k_Bacteria;p_Proteobacteria;c_Gammaproteobacteria;o_Burkholderiales;f_Burkholderiaceae;g_*Cupriavidus*;
*Psychrobacter*	0.016	0.01	0.01	0.01	0.01	0.01	0.02	0.00	0.00	k_Bacteria;p_Proteobacteria;c_Gammaproteobacteria;o_Pseudomonadales;f_Moraxellaceae;g_*Psychrobacter*;
*Ralstonia*	0.00	0.00	0.00	0.02	0.02	0.02	0.01	0.01	0.01	k_Bacteria;p_Proteobacteria;c_Gammaproteobacteria;o_Burkholderiales;f_Burkholderiaceae;g_*Ralstonia*;
*Bacteroides*	0.00	0.00	0.00	0.00	0.00	0.00	0.02	0.00	0.01	k_Bacteria;p_Bacteroidota;c_Bacteroidia; o_Bacteroidales;f_Bacteroidaceae;g_*Bacteroides*;
*Blautia*	0.00	0.00	0.00	0.00	0.00	0.00	0.00	0.00	0.01	k_Bacteria;p_Firmicutes;c_Clostridia; o_Lachnospirales;f_Lachnospiraceae;g_*Blautia*;
*Mitochondria*	0.00	0.01	0.00	0.00	0.00	0.00	0.00	0.00	0.00	k_Bacteria;p_Proteobacteria;c_Alphaproteobacteria; o_Rickettsiales;f_Mitochondria;g_*Mitochondria*;
*Bifidobacterium*	0.01	0.00	0.00	0.00	0.00	0.00	0.00	0.00	0.00	k_Bacteria;p_Actinobacteriota;c_Actinobacteria; o_Bifidobacteriales;f_Bifidobacteriaceae; g_*Bifidobacterium*;

**Table 4:** Alpha diversity statistics for Shannon, Simpson, Richness, Chao1, and ACE metrics in symbiotic microbiota of *D. magna* across generations from “BLX” and “CRP” ponds.

Sample	Shannon	Simpson	Richness	Chao1	ACE
BLX-G1.1	1.35	0.39	148	184.03	194.44
BLX-G1.2	1.29	0.38	126	151.2	157.62
BLX-G1.3	1.39	0.43	118	144.25	152.91
BLX-G4.1	3.53	0.94	164	166.28	168.73
BLX-G4.2	4.10	0.96	190	190	190
BLX-G4.3	3.45	0.93	153	157.4	157.64
BLX-G7.1	3.33	0.92	172	205.68	200.28
BLX-G7.2	3.43	0.92	212	248.03	249.21
BLX-G7.3	3.10	0.92	148	191.04	190.28
CRP-G1.1	2.73	0.78	189	254.03	262.01
CRP-G1.2	2.32	0.71	101	115	118.64
CRP-G1.3	2.61	0.76	170	190.32	194
CRP-G4.1	2.62	0.84	107	120.13	122.62
CRP-G4.2	2.74	0.86	112	143.07	143.68
CRP-G4.3	2.91	0.90	111	121	126.08
CRP-G7.1	2.57	0.82	146	193.3	183.77
CRP-G7.2	2.50	0.78	164	240	227.18
CRP-G7.3	2.80	0.83	187	229.43	242.41

## Data Availability

The original contributions presented in this study are included in the article/Supplementary Materials. Further inquiries can be directed to the corresponding author.

## Supplementary Materials

The following supporting information can be downloaded at: https://www.mdpi.com/article/10.3390/microorganisms12122492/s1, Table S1: Analysis of variance (ANOVA) tables of α-diversity including degrees of freedom, sums of squares, mean squares, F, *p*, and R values in symbiotic microbiota of *D. magna* from “BLX” and “CRP” ponds; Table S2: Permutational multivariate analysis of variance (PERMANOVA) tables of β-diversity including degrees of freedom, sums of squares, mean squares, F, *p*, and R values in symbiotic microbiota of *D. magna* from “BLX” and “CRP” ponds; Table S3: analysis of variance (ANOVA) tables of ASVs (relative abundance > 1%) including degrees of freedom, sums of squares, mean squares, F, *p*, and R values in symbiotic microbiota of *D. magna* from “BLX” pond; Table S4: Analysis of variance (ANOVA) tables of ASVs (relative abundance > 1%) including degrees of freedom, sums of squares, mean squares, F, *p*, and R values in symbiotic microbiota of *D. magna* from “CRP” pond.

## References

[1] Ezenwa V.O., Gerardo N.M., Inouye D.W., Medina M., Xavier J.B. (2012). Animal behavior and the microbiome. Science.

[2] Sommer F., Baeckhed F. (2013). The gut microbiota—Masters of host development and physiology. Nat. Rev. Microbiol..

[3] McFall-Ngai M., Hadfield M.G., Bosch T.C.G., Carey H.V., Domazet-Loso T., Douglas A.E., Dubilier N., Eberl G., Fukami T., Gilbert S.F. (2013). Animals in a bacterial world, a new imperative for the life sciences. Proc. Natl. Acad. Sci. USA.

[4] Shin S.C., Kim S.-H., You H., Kim B., Kim A.C., Lee K.-A., Yoon J.-H., Ryu J.-H., Lee W.-J. (2011). Drosophila microbiome modulates host developmental and metabolic homeostasis via insulin signaling. Science.

[5] Sison-Mangus M.P., Mushegian A.A., Ebert D. (2015). Water fleas require microbiota for survival, growth and reproduction. ISME J..

[6] Bang C., Dagan T., Deines P., Dubilier N., Duschl W.J., Fraune S., Hentschel U., Hirt H., Huelter N., Lachnit T. (2018). Metaorganisms in extreme environments: Do microbes play a role in organismal adaptation?. Zoology.

[7] Reshef L., Koren O., Loya Y., Zilber-Rosenberg I., Rosenberg E. (2006). The coral probiotic hypothesis. Environ. Microbiol..

[8] Kolodny O., Schulenburg H. (2020). Microbiome-mediated plasticity directs host evolution along several distinct time scales. Philos. Trans. R. Soc. B-Biol. Sci..

[9] Baldassarre L., Ying H., Reitzel A., Franzenburgq S., Fraune S. (2022). Microbiota mediated plasticity promotes thermal adaptation in the sea anemone *Nematostella vectensis*. Nat. Commun..

[10] Lampert W. (2011). Daphnia: Development of a Model Organism in Ecology and Evolution. Excellence in Ecology.

[11] Bekker E.I., Karabanov D.P., Galimov Y.R., Haag C.R., Neretina T.V., Kotov A.A. (2018). Phylogeography of *Daphnia magna* Straus (Crustacea: Cladocera) in Northern Eurasia: Evidence for a deep longitudinal split between mitochondrial lineages. PLoS ONE.

[12] Fields P.D., Reisser C., Dukic M., Haag C.R., Ebert D. (2015). Genes mirror geography in *Daphnia magna*. Mol. Ecol..

[13] Ma X., Ni Y., Wang X., Hu W., Yin M. (2020). Lineage diversity, morphological and genetic divergence in *Daphnia magna* (Crustacea) among Chinese lakes at different altitudes. Contrib. Zool..

[14] Peerakietkhajorn S., Kato Y., Kasalicky V., Matsuura T., Watanabe H. (2016). Betaproteobacteria limnohabitans strains increase fecundity in the crustacean *Daphnia magna*: Symbiotic relationship between major bacterioplankton and zooplankton in freshwater ecosystem. Environ. Microbiol..

[15] Mushegian A.A., Walser J.-C., Sullam K.E., Ebert D. (2018). The microbiota of diapause: How host-microbe associations are formed after dormancy in an aquatic crustacean. J. Anim. Ecol..

[16] Cooper R.O., Cressler C.E. (2020). Characterization of key bacterial species in the *Daphnia magna* microbiota using shotgun metagenomics. Sci. Rep..

[17] Macke E., Callens M., De Meester L., Decaestecker E. (2017). Host-genotype dependent gut microbiota drives zooplankton tolerance to toxic cyanobacteria. Nat. Commun..

[18] Hegg A., Radersma R., Uller T. (2021). A field experiment reveals seasonal variation in the *Daphnia* gut microbiome. Oikos.

[19] Thompson L.G., Yao T., Mosley-Thompson E., Davis M.E., Henderson K.A., Lin P.N. (2000). A high-resolution millennial record of the South Asian Monsoon from Himalayan ice cores. Science.

[20] Clewing C., Albrecht C., Wilke T. (2016). A complex system of Glacial sub-Refugia drives endemic freshwater biodiversity on the Tibetan Plateau. PLoS ONE.

[21] Niu Y., Yang S., Zhou J., Chu B., Ma S., Zhu H., Hua L. (2019). Vegetation distribution alongmountain environmental gradient predicts shifts in plant community response to climate change in alpine meadow on the Tibetan Plateau. Sci. Total Environ..

[22] Folmer O.F., Black M.B., Hoeh W.R., Lutz R.A., Vrijenhoek R.C. (1994). DNA primers for amplification of mitochondrial cytochrome c oxidase subunit I from diverse metazoan invertebrates. Mol. Mar. Biol. Biotechnol..

[23] Kilham S.S., Kreeger D.A., Lynn S.G., Goulden C.E., Herrera L. (1998). COMBO: A defined freshwater culture medium for algae and zooplankton. Hydrobiologia.

[24] Pichler M., Coskun O.K., Ortega-Arbulu A.-S., Conci N., Woerheide G., Vargas S., Orsi W.D. (2018). A 16S rRNA gene sequencing and analysis protocol for the Illumina MiniSeq platform. Microbiologyopen.

[25] Callahan B.J., McMurdie P.J., Rosen M.J., Han A.W., Johnson A.J.A., Holmes S.P. (2016). DADA2: High-resolution sample inference from Illumina amplicon data. Nat. Methods.

[26] Bolyen E., Rideout J.R., Dillon M.R., Bokulich N.A., Abnet C.C., Al-Ghalith G.A., Alexander H., Alm E.J., Arumugam M., Asnicar F. (2019). Reproducible, interactive, scalable and extensible microbiome data science using QIIME 2 (vol 37, pg 852, 2019). Nat. Biotechnol..

[27] Costantini M.S., Medeiros M.C.I., Crampton L.H., Reed F.A. (2021). Wild gut microbiomes reveal individuals, species, and location as drivers of variation in two critically endangered Hawaiian honeycreepers. PeerJ.

[28] Locher K., Belanger C.R., Eckbo E., Caza M., Velapatino B., Charles M.K. (2022). Automated 16S Sequencing Using an R-Based Analysis Module for Bacterial Identification. Microbiol. Spectr..

[29] McMurdie P.J., Holmes S. (2013). phyloseq: An R Package for Reproducible Interactive Analysis and Graphics of Microbiome Census Data. PLoS ONE.

[30] Segata N., Izard J., Waldron L., Gevers D., Miropolsky L., Garrett W.S., Huttenhower C. (2011). Metagenomic biomarker discovery and explanation. Genome Biol..

[31] Webster N.S., Reusch T.B.H. (2017). Microbial contributions to the persistence of coral reefs. ISME J..

[32] van Oppen M.J.H., Blackall L.L. (2019). Coral microbiome dynamics, functions and design in a changing world. Nat. Rev. Microbiol..

[33] Alberdi A., Aizpurua O., Bohmann K., Zepeda-Mendoza M.L., Gilbert M.T.P. (2016). Do vertebrate gut metagenomes confer rapid ecological adaptation?. Trends Ecol. Evol..

[34] Qi W., Nong G., Preston J.F., Ben-Ami F., Ebert D. (2009). Comparative metagenomics of *Daphnia* symbionts. BMC Genom..

[35] Callens M., Watanabe H., Kato Y., Miura J., Decaestecker E. (2018). Microbiota inoculum composition affects holobiont assembly and host growth in *Daphnia*. Microbiome.

[36] Akbar S., Huang J., Zhou Q., Gu L., Sun Y., Zhang L., Lyu K., Yang Z. (2021). Elevated temperature and toxic Microcystis reduce *Daphnia* fitness and modulate gut microbiota. Environ. Pollut..

[37] Peerakietkhajorn S., Tsukada K., Kato Y., Matsuura T., Watanabe H. (2015). Symbiotic bacteria contribute to increasing the population size of a freshwater crustacean, *Daphnia magna*. Environ. Microbiol. Rep..

[38] Gorokhova E., Rivetti C., Furuhagen S., Edlund A., Ek K., Breitholtz M. (2015). Bacteria-mediated effects of antibiotics on *Daphnia* nutrition. Environ. Sci. Technol..

[39] Pladdies T., Babenzien H.D., Cypionka H. (2004). Distribution of *Nevskia ramosa* and other rosette-forming neustonic bacteria. Microb. Ecol..

[40] Sturmeyer H., Overmann J., Babenzien H.D., Cypionka H. (1998). Ecophysiological and phylogenetic studies of *Nevskia ramosa* in pure culture. Appl. Environ. Microbiol..

[41] Wotton R.S., Preston T.M. (2005). Surface films: Areas of water bodies that are often overlooked. Bioscience.

[42] Zhang J., Xiao Y., Wang H., Zhang H., Chen W., Lu W. (2023). Lactic acid bacteria-derived exopolysaccharide: Formation, immunomodulatory ability, health effects, and structure-function relationship. Microbiol. Res..

[43] Zikmanis P., Brants K., Kolesovs S., Semjonovs P. (2020). Extracellular polysaccharides produced by bacteria of the Leuconostoc genus. World J. Microbiol. Biotechnol..

[44] Rastogi S., Singh A. (2022). Gut microbiome and human health: Exploring how the probiotic genus *Lactobacillus* modulate immune responses. Front. Pharmacol..

[45] Capurso L. (2019). Thirty Years of *Lactobacillus rhamnosus* GG: A Review. J. Clin. Gastroenterol..

[46] Chiriac M.-C., Haber M., Salcher M.M. (2023). Adaptive genetic traits in pelagic freshwater microbes. Environ. Microbiol..

[47] Compant S., Nowak J., Coenye T., Clement C., Barka E.A. (2008). Diversity and occurrence of *Burkholderia* spp. in the natural environment. FEMS Microbiol. Rev..

[48] Nowak J., Shulaev V. (2003). Priming for transplant stress resistance in in vitro propagation. In Vitro Cell. Dev. Biol.-Plant.

[49] O’Sullivan L.A., Weightman A.J., Jones T.H., Marchbank A.M., Tiedje J.M., Mahenthiralingam E. (2007). Identifying the genetic basis of ecologically and biotechnologically useful functions of the bacterium *Burkholderia vietnamiensis*. Environ. Microbiol..

[50] Depoorter E., Bull M.J., Peeters C., Coenye T., Vandamme P., Mahenthiralingam E. (2016). *Burkholderia*: An update on taxonomy and biotechnological potential as antibiotic producers. Appl. Microbiol. Biotechnol..

[51] Lindstrom E.S., Langenheder S. (2012). Local and regional factors influencing bacterial community assembly. Environ. Microbiol. Rep..

[52] Eckert E.M., Anicic N., Fontaneto D. (2021). Freshwater zooplankton microbiome composition is highly flexible and strongly influenced by the environment. Mol. Ecol..

[53] Callens M., De Meester L., Muylaert K., Mukherjee S., Decaestecker E. (2020). The bacterioplankton community composition and a host genotype dependent occurrence of taxa shape the *Daphnia magna* gut bacterial community. FEMS Microbiol. Ecol..

[54] Frankel-Bricker J., Song M.J., Benner M.J., Schaack S. (2020). Variation in the microbiota associated with *Daphnia magna* across genotypes, populations, and temperature. Microb. Ecol..

[55] Sullam K.E., Pichon S., Schaer T.M.M., Ebert D. (2018). The combined effect of temperature and host clonal line on the microbiota of a planktonic Crustacean. Microb. Ecol..

[56] Akbar S., Li X., Ding Z., Liu Q., Huang J., Zhou Q., Gu L., Yang Z. (2022). Disentangling diet- and medium-associated microbes in shaping *Daphnia* gut microbiome. Microb. Ecol..

