# Supplementary material for: Environmental Changes Driving Shifts in the Structure and Functional Properties of the Symbiotic Microbiota of Daphnia
Source: Microorganisms. 2024 Dec 3;12(12):2492. doi: 10.3390/microorganisms12122492 (PMC11728151; doi:10.3390/microorganisms12122492)
Supplement: Supplementary file 1 [file microorganisms-12-02492-s001.zip › microorganisms-3336798-supplementary.pdf]

Table S1. ANOVA tables of α-diversity including degrees of freedom, sums of squares, mean squares, F, *p* and R values in the symbiotic microbiota of *D. magna* from "BLX" and "CRP" pond.

	Degrees of freedom	Sum of squares	Mean square	F	*p*	R
BLX Shannon	2	9.51	4.75	91.87	<0.0001	0.97
CRP Shannon	2	0.06	0.03	1.04	0.41	0.26
BLX ACE	2	3725	1863	3.14	0.12	0.51
CRP ACE	2	11948	5974	2.89	0.13	0.49

Table S2. PERMANOVA tables of β-diversity including degrees of freedom, sums of squares, mean squares, F, *p* and R values in the symbiotic microbiota of *D. magna* from "BLX" and "CRP" pond.

	Degrees of freedom	Sum of squares	Mean square	F	*p*	R
BLX	2	0.26	0.13	90.89	0.004	0.97
CRP	2	0.22	0.11	36.02	0.005	0.92

Table S3. ANOVA tables of ASVs (relative abundance>1%) including degrees of freedom, sums of squares, mean squares, F, *p* and R values in the symbiotic microbiota of *D. magna* from "BLX" pond.

	Degrees of freedom	Sum of squares	Mean square	F	*p*	R
Nevskia	2	0.5828	0.2914	851.2	<0.0001	0.9965
Blastomonas	2	0.01563	0.007816	5100	<0.0001	0.9994
Vibrio	2	0.01169	0.005845	67.23	<0.0001	0.9573
Acidovorax	2	0.001674	0.0008368	1	0.4219	0.25
Perlucidibaca	2	0.004284	0.002142	69.31	<0.0001	0.9585
Emticicia	2	0.003633	0.001816	98.6	<0.0001	0.9705
Pseudomonas	2	0.0006889	0.0003444	4.275	0.0701	0.5876
NS11-12_marine_group	2	0.002305	0.001153	2883	<0.0001	0.999
Rhodoferax	2	0.0002492	0.0001246	1	0.4219	0.25
Leuconostoc	2	0.0006887	0.0003443	3.736	0.0883	0.5546
Pseudoalteromonas	2	0.0011	0.0005498	105.4	<0.0001	0.9723
Lactobacillus	2	0.0003917	0.0001958	3.29	0.1085	0.5231
Acinetobacter	2	0.0006242	0.0003121	10.49	0.011	0.7776
Pedobacter	2	0.0004301	0.0002151	7.89	0.0209	0.7245
Limnobacter	2	0.0004924	0.0002462	433.8	<0.0001	0.9931
Rhodanobacter	2	0.0003882	0.0001941	2075	<0.0001	0.9986
Pirellula	2	3.85E-05	1.92E-05	1	0.4219	0.25
Allorhizobium-Neo	2	3.68E-05	1.84E-05	1	0.4219	0.25
Muribaculaceae	2	2.64E-05	1.32E-05	1	0.4219	0.25

Table S4. ANOVA tables of ASVs (relative abundance>1%) including degrees of freedom, sums of squares, mean squares, F, *p* and R values in the symbiotic microbiota of *D. magna* from "CRP" pond.

	Degrees of freedom	Sum of squares	Mean square	F	*p*	R
Nevskia	2	0.1763	0.08813	136.9	<0.0001	0.9786
Lacihabitans	2	0.07779	0.03889	40.25	0.0003	0.9306
Pirellula	2	0.047	0.0235	383.4	<0.0001	0.9922
Limnobacter	2	0.036	0.018	164	<0.0001	0.982
Sphingomonas	2	0.008857	0.004428	40.03	0.0003	0.9303
Mycoplasma	2	0.0017	0.0008502	14.14	0.0054	0.825
Escherichia-Shigella	2	0.0005586	0.0002793	2.057	0.2088	0.4067
Pseudomonas	2	0.001315	0.0006574	23.06	0.0015	0.8849
Vibrio	2	0.001007	0.0005033	22.78	0.0016	0.8836
Methylophilus	2	0.001513	0.0007564	45.52	0.0002	0.9382
Chloroplast	2	0.0002402	0.0001201	1	0.4219	0.25
FukuN57	2	0.001067	0.0005337	27	0.001	0.9
Nitrosomonas	2	0.0001347	6.74E-05	1	0.4219	0.25
Cupriavidus	2	0.0003821	0.0001911	8.145	0.0195	0.7308
Psychrobacter	2	0.0001095	5.47E-05	1.144	0.3794	0.2761
Ralstonia	2	0.0004369	0.0002184	269.5	<0.0001	0.989
Bacteroides	2	0.0001773	8.86E-05	3.797	0.086	0.5586
Blautia	2	4.10E-05	2.05E-05	1	0.4219	0.25
Mitochondria	2	3.82E-05	1.91E-05	1	0.4219	0.25
Bifidobacterium	2	2.99E-05	1.50E-05	1	0.4219	0.25
